# Omega-3 and omega-6 content of medicinal foods for depressed patients: implications from the Iranian Traditional Medicine

**Published:** 2014

**Authors:** Mandana Tavakkoli-Kakhki, Malihe Motavasselian, Mahmoud Mosaddegh, Mohammad Mahdi Esfahani, Mohammad Kamalinejad, Mohsen Nematy, Saeid Eslami

**Affiliations:** 1 *Department of Traditional Medicine, School of Traditional Medicine, Shahid Beheshti University of Medical Sciences, Tehran, I. R. Iran*; 2 *Department of Traditional Medicine, Faculty of Traditional Medicine, Tehran University of Medical Sciences, Tehran, I. R. Iran*; 3 *Department of Pharmacognosy, Faculty of Pharmacy, Shahid Beheshti University of Medical Sciences, Tehran, I. R. Iran*; 4 *Department of Nutrition, School of Medicine, Biochemistry and Nutrition, Endoscopic and Minimally Invasive Surgery and Cancer Research Centers, Mashhad University of Medical Sciences, Mashhad, I. R. Iran*; 5 *Pharmaceutical Research Center, School of Pharmacy, Mashhad University of Medical Sciences, Mashhad, I. R. Iran*; 6 *Department of Medical Informatics, Academic Medical Center, University of Amsterdam, Amsterdam, the Netherlands*

**Keywords:** *Depression*, *Fatty acids*, *Functional food*, *Medicine*, *Omega-3*, *Traditional*

## Abstract

**Objectives:** Considering the increasing prevalence of depression in modern societies and the positive effects of omega-3 polyunsaturated fatty acids on depression, this study aims to investigate the omega-3 and omega-6 content of various foodstuffs, prescribed or prohibited by Iranian Traditional Medicine (ITM).

**Materials and Methods:** Firstly, reliable sources of Iranian Traditional Medicine were reviewed in order to identify the prescribed and prohibited foodstuffs for depressed patients. Afterwards, according to the online database of United States Department of Agriculture (URL: http://ndb.nal.usda.gov/ndb/search/list), the ratio of linoleic acid to alpha linolenic acid (as representatives of omega-6 and omega-3, respectively) was identified in each foodstuff. Finally, the ratios of omega-6 to omega-3 were compared between seven food groups of vegetables, fruits, dry goods, high protein products, dairies, breads, and spices.

**Results: **Based on the resources of Iranian Traditional Medicine, the following foods are prescribed for depressed patients: basil, coriander, spinach, lettuce, squash, peppermint, dill, chicory, celery, beet, quince, cucumber, watermelon, grape, peach, pomegranate, banana, apple, currant, pistachio, dried fig, almond, egg, chicken, lamb, trout, milk, bread without bran, saffron, oregano, and coriander seeds. On the other hand, cabbage, eggplant, onion, garlic, broad beans, lentils, beef, whole wheat bread, and mustard are prohibited. It should be noted that omega-3 content in some prescribed foods is more than that of the prohibited ones.

**Conclusion: **The present study showed that mint, basil, spinach, lettuce, squash, lamb, saffron, oregano, cucumber, pistachio, milk, and also wild trout can be considered as medicinal foods for depressed patients.

## Introduction

Recently, a great deal of attention has been paid to the use of therapeutic methods for depression, based on Complementary and Alternative Medicine (CAM) (Sadock et al., 2007 ▶). This is due to the high prevalence of mood disorders in today’s modern societies (Richards, 2011 ▶), side effects of long-term medications (Sadock et al., 2007 ▶), and significant complications associated with depression such as the increased risk of cardiovascular diseases (Nemeroff and Goldschmidt-Clermont, 2012 ▶), Alzheimer’s disease (Wint, 2011 ▶), and diabetes (Silva et al., 2012 ▶). 

Iranian Traditional Medicine (ITM) as a CAM involves several non-pharmacological treatments, among which food therapy is the most notable. Although the effectiveness of different diets, such as the Mediterranean diet, in preventing depression (Sánchez-Villegas et al., 2006 ▶) and reducing the related complications (Antonogeorgos et al., 2012 ▶) has been approved, studies which specifically introduce the effective foodstuffs are quite limited. Detailed studies on ITM texts indicated that some foodstuffs are prescribed and some others are prohibited for melancholia management. Based on ITM resources, melancholia is considered as a psychotic type of depression (Ahwazi Arjani, 1973 ▶; Ibn Sina, 2005 ▶; JorJani, 1976 ▶). 

Considering the positive effects of omega-3 fatty acids on depression management (Appleton et al., 2010 ▶; Kraguljac et al., 2009 ▶), this paper intends to investigate the prescribed and prohibited foodstuffs, with a focus on their omega-3 content. 

## Materials and Methods

At first, reliable ITM texts including Kamel al-Sanaat al-Tibbyyah, Al-Qanun fi al-Tibb and Zakhireh Kharazmshahi were reviewed for prescribed and prohibited foods in depression. Given to the positive impacts of polyunsaturated fatty acids on depressed patients and for investigating omega-3 content of the foodstuffs, in the next step the amounts of omega-3 and also omega-6 polyunsaturated fatty acids for each of the foodstuffs were collected from the USDA database (United States Department of Agriculture, URL: http://ndb.nal.usda.gov/ndb/search/list, 2011). It should be noted that the values of linoleic acid and alpha linolenic acid are considered relative to the amount of omega-6 and omega-3, respectively. In the next step, the ratio of omega-6 to omega-3 was determined for each of the foodstuffs in seven food groups, including vegetables, fruits, dry goods, high protein products, dairies, breads, and spices. Finally, in each of the aforementioned groups, the obtained ratios were compared with each other. 

It should be noted that for the vegetables group, high protein products, breads, and spices, the comparison was made between the prescribed and prohibited foodstuffs. On the other hand, for the two groups of fruits and dry goods, the comparison was made between the prescribed foodstuffs, since they were the only ones mentioned in the resources.

## Results

Based on the available evidence in the reliable ITM texts, using basil, coriander, spinach, lettuce, squash, peppermint, dill, chicory, celery, beet, quince, cucumber, watermelon, grape, peach, pomegranate, banana, apple, currant, pistachio, dried fig, almond, egg, chicken, lamb, trout, milk, bread without bran, saffron, oregano, and coriander seeds is prescribed for depression management. On the other hand, using cabbage, eggplant, onion, garlic, broad beans, lentils, beef, whole wheat bread, and mustard is prohibited (Ahwazi Arjani, 1973 ▶; Ibn-e Sina, 2005 ▶; JorJani, 1976 ▶).

With regard to the values of omega-3 and omega-6 polyunsaturated fatty acids obtained from the USDA database (United States Department of Agriculture, URL: http://ndb.nal.usda.gov/ndb/search/list, 2011), in the vegetables group, the ratio of omega-6 to omega-3 in mint, spinach, basil, lettuce, and squash is less in comparison with all the prohibited vegetables. However, this does not apply to coriander, dill, chicory, celery, and beet, which are prescribed in this group (See Table 1).

**Table 1:** An approximate ratio of omega-6 PUFA to omega-3 PUFA in each of prohibited and prescribed foodstuffs

**Food groups**	**Subgroups**	**Foodstuffs**	**Omega-6 PUFA(g)**	**Omega-3 PUFA(g)**	**Omega-6 PUFA/Omega-3 PUFA**
**Vegetables**	Prescribed vegetables	Coriander	0.04	0	∞
		Dill	0.082	0.013	6/1
		Basil	0.073	0.316	1/4
		Peppermint	0.069	0.435	1/7
		Celery	0.079	0	∞
		Squash	0.016	0.026	1/2
		Lettuce	0.024	0.058	1/2
		Chicory	0.112	0.019	6/1
		Beet	0.055	0.005	11/1
		Spinach	0.026	0.138	1/5
	Prohibited vegetables	Cabbage	0.017	0	∞
		Garlic	0.229	0.02	11/1
		Onions	0.013	0.004	3/1
		Eggplant	0.063	0.013	5/1
**Prescribed dried fruits and nuts**		Currant	0.18	0	∞
		Pistachio	13.485	0.259	65/1
		Almond	12.061	0.006	2000/1
		Dried fig	0.345	0	∞
**Prescribed fruits**		Quince	0.049	0	∞
		Watermelon	0.05	0	∞
		Peach	0.084	0.002	42/1
		Banana	0.046	0.027	2/1
		Apple	0.043	0.009	5/1
		Cucumber	0.002	0.002	1/1
		Grape	0.037	0.011	3/1
		Pomegranate	0.079	0	∞
**High protein products**	Prescribed high protein products	Trout	0.492	0.067	8/1
		Lamb	1.12	0.33	3/1
		Chicken	3.86	0.18	20/1
		Egg	1.555	0.048	31/1
	Prohibited high protein products	Beef	0.42	0.21	2/1
		Lentils	0.404	0.109	4/1
		Broad beans	0.581	0.046	12/1
**Spices**	Prescribed spices	Coriander seeds	1.75	0	∞
		Oregano	0.74	0.62	1.16/1
		Saffron	0.75	1.24	0.58/1
	Prohibited spices	Mustard	5.92	3.79	1.5/1

In the group of fruits, the ratio of omega-6 to omega-3 in cucumber is less than that of other prescribed fruits (See Table 1). In the group of dried fruits and nuts, the ratio for pistachio is less than that of other prescribed dried fruits and nuts (Table 1).

Regarding high protein products, the ratio of omega-6 to omega-3 in lamb is less than that of broad beans and lentils. However, this does not apply to egg, chicken, and trout**, **which are prescribed in this group. Contrary to our expectations, this ratio is less for the beef in comparison with the lamb (Table 1). The ratio of omega-6 to omega-3 in milk, prescribed in the group of dairy products, is 1.6:1. Considering the group of spices, the ratio in saffron and oregano is less than that of mustard, but such a characteristic is not found in the prescribed coriander seeds (Table 1). In the group of breads, the ratio of omega-6 to omega-3 in bread without bran (8.5:1) is less than that of whole wheat bread (9:1).

## Discussion

For the interpretation of the obtained results, it is assumed that the desired result for each of the mentioned food groups is obtained if the ratio of omega-6 to omega-3 in a prescribed foodstuff is less than that of all the prohibited ones, in the same group. Moreover, based on our presumptions, if this ratio in a prescribed foodstuff reduces, the food can be more suitable for depressed patients. It should be noted that the optimal omega-6 to omega-3 ratio is estimated to be 2:1 to 3:1, which is four times less than the usual daily intake (Mahan and Stump, 2008 ▶).

Accordingly, the obtained results were not favorable in regard to certain prescribed foodstuffs including some vegetables such as coriander, dill, chicory, celery, and beef as well as some high protein products such as egg, chicken, trout, and coriander seeds in the prescribed spices. 

However, the desired result is obtained for mint, basil, spinach, lettuce, and squash in the group of vegetables, lamb in the group of high protein products, saffron and oregano in the group of spices, and finally white bread in the group of breads. In regard to the groups of fruits and dried fruits, it may be concluded that cucumber and pistachio are the most effective fruits and dried fruits, respectively. For milk which is a prescribed dairy product, the ratio of linoleic acid to alpha linolenic acid is 1.6:1, which is close to the optimal ratios. Therefore, in terms of omega-3 content, mint, basil, spinach, lettuce, squash, lamb, saffron, oregano, white bread, cucumber, pistachio, and milk are the most effective foodstuffs for depressed patients.

Some studies have been conducted on the health benefits of fish as a source of omega-3 fatty acids for depression (Bountziouka et al., 2009 ▶; Li et al., 2011 ▶; Murakami et al., 2010 ▶; Su, 2009 ▶; Suominen-Taipale et al., 2010 ▶; Timonen et al., 2004 ▶). 

Contrary to our expectations, the ratio of omega-6 to omega-3 in trout (8:1) was higher than those of lamb (3:1) and beef (2:1). Therefore, based on our findings, beef and lamb are preferable to trout, with regard to their omega-3 content. Since the approximate ratio of omega-6 to omega-3 in wild trout is 2:1, this contradiction could be related to selecting framed trout instead of wild trout in this study.

Recent studies show the negative effect of non-bran grain consumption on depression (Jacka, 2010 ▶), while omega-3 content in white bread is more than that of whole bread. This contradiction may be resolved considering to prevalence of constipation in modern societies. In the past, constipation was not as prevalent as it is today, whereas nowadays, it is considered to be an important issue (Ford and Suares, 2011). Therefore, although white bread has more omega-3 content, but with regard to the positive effect of fibers on constipation and subsequent depression (Kathleen Mahan and Escott-Stump, 2008; Logan, 2006 ▶), whole bread may be considered as a preferable choice in the current societies.

On the other hand, modern medicine databases have reported the antidepressant effect of garlic (Dhingra and Kumar, 2008 ▶), whereas based on our findings, it is a poor vegetable in terms of omega-3 content.

In the present study, we studied only the USDA database for polyunsaturated fatty acids values, while reports by other resources were neglected. Because this database is one of the most valuable and well-known resources in this domain, the obtained results are most likely reliable.

Other limitation of this study is that the effects of other nutrients have not been considered. Omega-3 is only one of the effective nutrients for depression management and there are many other nutrients that have positive effect on depression. Therefore, it is not possible to confirm the antidepressant effects of foodstuffs only by relying on their fatty acids content. 

As the final point, mint, basil, spinach, lettuce, squash, lamb, saffron, oregano, cucumber, pistachio, milk, and also wild trout can be considered as medicinal foods for depressed patients. On the other hand, cabbage, eggplant, onion, broad beans, lentils, beef, and mustard do not have such effect. By definition, medicinal foods not only provide the required nutrients, but also promote the health status of an individual (Esfahani et al., 2011 ▶). 

As previously mentioned, polyunsaturated fatty acids are only part of the nutrients affecting depression and nutrients are only part of the effective ingredients in a foodstuff. Accordingly, research on the effects and amounts of other nutrients in the studied foodstuffs would be helpful in achieving more accurate results. Moreover, it would be advantageous to design observational or interventional studies based on the main findings of this study.
